# Fast Distributed Model Predictive Control Method for Active Suspension Systems

**DOI:** 10.3390/s23063357

**Published:** 2023-03-22

**Authors:** Niaona Zhang, Sheng Yang, Guangyi Wu, Haitao Ding, Zhe Zhang, Konghui Guo

**Affiliations:** 1 School of Electrical and Electronic Engineering, Changchun University of Technology, Changchun 130012, China; 2 State Key Laboratory of Automobile Simulation and Control, Jilin University, Changchun 130012, China

**Keywords:** active suspension system, distributed model predictive control, multi-agent, RBF neural network

## Abstract

In order to balance the performance index and computational efficiency of the active suspension control system, this paper offers a fast distributed model predictive control (DMPC) method based on multi-agents for the active suspension system. Firstly, a seven-degrees-of-freedom model of the vehicle is created. This study establishes a reduced-dimension vehicle model based on graph theory in accordance with its network topology and mutual coupling constraints. Then, for engineering applications, a multi-agent-based distributed model predictive control method of an active suspension system is presented. The partial differential equation of rolling optimization is solved by a radical basis function (RBF) neural network. It improves the computational efficiency of the algorithm on the premise of satisfying multi-objective optimization. Finally, the joint simulation of CarSim and Matlab/Simulink shows that the control system can greatly minimize the vertical acceleration, pitch acceleration, and roll acceleration of the vehicle body. In particular, under the steering condition, it can take into account the safety, comfort, and handling stability of the vehicle at the same time.

## 1. Introduction

The pitch and roll motions of the vehicle will cause the occupants to shake, which seriously affects the ride comfort of the vehicle. Therefore, research on the restraint of the pitch and roll motion of the vehicle has great practical significance [1,2]. With the gradual intellectualization, networking, electrification, and sharing of the automotive technology field, people have higher demands for computing power, ride comfort, and driving safety.

Compared with passive and semi-active suspension, active suspension reduces the vibration of sprung mass caused by road excitation in an active way, so the damping effect is more obvious [3]. Active suspension can isolate the road vibration and enhance the road grip better under the control of its controller, which can not only enhance the comfort of the passengers but also ensure the safety of the vehicle. In addition, active suspension can realize multi-objective control, thus balancing the conflict between ride comfort and driving safety in electric vehicles [4,5].

At present, the more common control methods to improve suspension performance include optimal control [6], neural network control [7], adaptive control [8], sliding mode control [9,10], fuzzy control [11], and model predictive control [12,13,14]. Ding et al. [6] proposed the optimal selection strategy of anti-interference coefficients in the time-delay-dependent H-infinity/H-2 controller, and the effectiveness of the proposed method is verified by simulation. Wang et al. [7] proposed an output feedback algorithm based on a neural network for the active suspension system. They constructed an auxiliary system to compensate for the input saturation constraint, and riding comfort and safety conditions were ensured. Hao et al. [8] presented a novel multi-objective command-filtered adaptive control strategy for active suspension systems with nonlinear hydraulic actuators, which effectively improves the ride comfort. Control methods such as PID and LQR cannot provide the best effect for improving vehicle vibration; Chen et al. [9] proposed a revised active disturbance rejection sliding mode controller to improve the vertical stability of UGV. Liu et al. [10] proposed an adaptive sliding mode control method for active suspension systems with specified performance, which can stabilize the suspension system′s displacement and speed in finite time. Robert et al. [11] developed fuzzy control of active suspension system, and the results obtained from the simulation of the road profile show that the proposed fuzzy control performs better than the conventional controller in terms of body displacement and body acceleration. For the past few years, MPC has been widely studied by many scholars in dealing with large and complex systems, such as online processing of system state, output and control input constraints, high flexibility and fault tolerance, model dimensionality reduction, computation reduction, and control efficiency improvement. Myron et al. [12] presented a model predictive controller combined with radial basis function networks for the active suspension system, which demonstrated excellent performance in all scenarios when compared with passive suspension. Mai et al. [13] presented an explicit model predictive control method for the semi-active suspension system with magnetorheological dampers subject to input constraints, which effectively improved the comfort of a semi-active suspension system. The team led by Yu [14] designed a road preview model predictive control scheme for the semi-active suspension system with the magneto-rheological damper to improve the comprehensive performance of the semi-active suspension. In addition to some individuals, there are also many teams studying integrated control. In order to address both braking safety and ride comfort, Zhang et al. [15] established a comfort braking dynamics model for brake-by-wire vehicles, taking into account the relationship between braking and suspension dynamics. Liang et al. [16] proposed a decentralized cooperative control framework to achieve the integration of the active front steering system and the active suspension system by applying a multi-constrained distributed model predictive control approach.

The constrained optimization control capability of MPC is mainly produced by solving constrained quadratic programming (QP) problems online. Although the traditional QP numerical algorithm has been widely used, it involves matrix inversion, which results in the disadvantage of MPC in terms of solution speed. Yannic et al. [17] presented an optimal control strategy for the high computational requirements of nonlinear model predictive control by learning through artificial neural networks to speed up the computation while obtaining good objective function values and satisfying constraints. In the framework of a multi-agent network, Le et al. [18] proposed a collective neural dynamics optimization method based on a recurrent neural network to solve the control method of a distributed convex optimization problem, which avoids the calculation of matrix inversion and improves the execution efficiency of the algorithm. Wysocki et al. [19] have given an improved recurrent Elman neural network algorithm that can consider the time delay of the process and provide an MPC for the network.

A Multi-Agent System (MAS) is a group of agents that can work together to compute. Each agent completes tasks or reaches goals by working with other agents. MAS refers to a set composed of multiple agents that can perform network computing, in which agent completes tasks or achieves specific objectives through cooperation. It has been widely used in the automotive field [20]. Based on multi-agent theory, Zhang et al. [21] decomposed a four-wheel independent drive ASR system into four separate driving wheel agent systems. For actuator faults, a Lyapunov function based on multiagent theory was designed for a single driving wheel agent to avoid the impact of the coupling subsystem fault. Wang et al. [22] proposed a multi-objective optimization coordinated control method for ABS and AFS based on multi-agent MPC, and improved the braking safety and handling stability of the vehicle. Zhang et al. [23] presented a four-wheel independent steering finite time control method based on the theory of heterogeneous multi-agent, and the simulation results verify that the proposed method can improve the yaw stability of the vehicle. The four suspensions in the active suspension control system are scattered at the four wheels, and their communication is realized through the on-board CAN bus, which enables the signals transmitted on one data line to be shared by multiple control units (systems).

In particular, based on the multi-agent theory and neural network fast partial differential equation solving idea, this paper regards the body′s vertical, pitch, roll, and the vertical motion of the four wheels as seven agents. By using the mutual communication among the agents, a distributed model predictive control method of active suspension for engineering applications is proposed, which can improve the computational efficiency of the algorithm under the premise of satisfying multi-objective optimization. The contributions of this study are as follows:

(1) According to the dynamic mechanism of the vehicle and the working principle of the active suspension control system, by redefining the control input and constraints, the seven subsystems of the seven-degree-of-freedom vehicle model are regarded as seven agents, and the graph-theory-based active suspension dimensionality reduction control model is used to simplify the model dimension.

(2) Considering the influence of the state of other adjacent agents on its own agents, a system control model based on multi-agents is established, and the vertical vibration acceleration of the unsprung mass and the vertical acceleration of the vehicle body are realized through the cooperation between the agents. Body roll angular acceleration and body pitch angular acceleration follow their ideal values.

(3) In the model predictive control algorithm, the advantages of the simple structure and global approximation capability of the RBF neural network are used to propose a fast optimal solution method for the *i*-th intelligent body based on the RBF neural network to quickly find the rolling optimal solution in the model predictive control algorithm.

**Figure 1:**
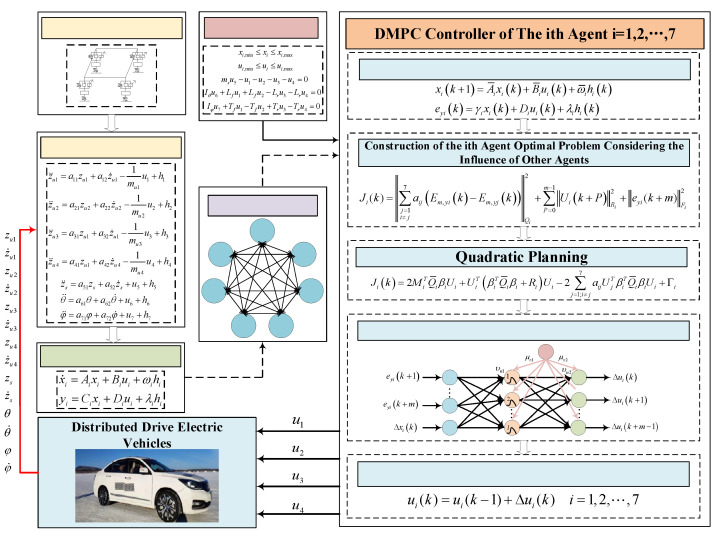
Overall framework of the paper.

The rest of this article is described as follows: In the second segment, a seven-degrees-of-freedom vehicle model is established. In the third segment, in order to comprehensively analyze the performance of the suspension according to its network topology and mutual coupling constraints, a vehicle model is established based on graph theory with reduced dimensionality. In the fourth segment, a multi-agent-based distributed model predictive controller is designed. The RBF neural network is used to improve the solution speed of partial differential equations, and the effectiveness of the proposed method is verified by simulation. Finally, the fifth segment draws conclusions. The overall framework of this paper is shown in Figure 1.

## 2. Seven-DOF Vehicle Model

**Figure 2:**
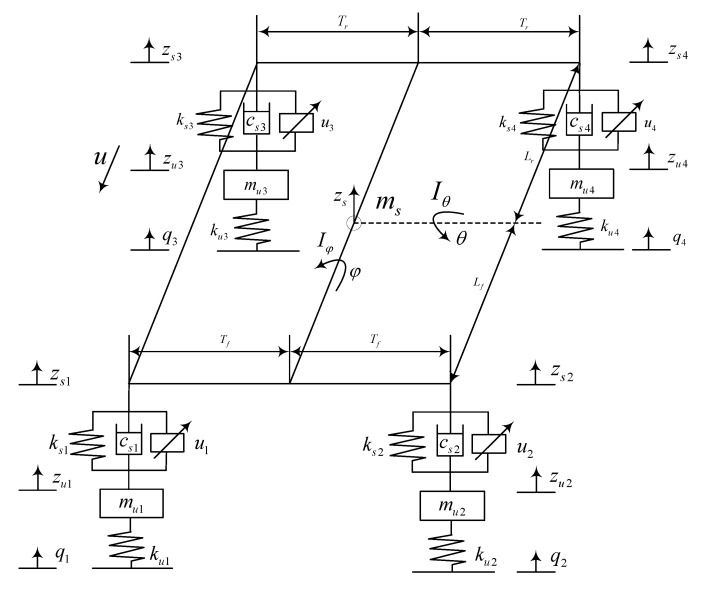
**Seven**-DOF Model of the Full Vehicle.

At present, in the research of active suspension control, the 1/4 vehicle model, the 1/2 vehicle model, and the whole vehicle model are the research objects [24,25,26]. Using the two-degrees-of-freedom model as the object of study can better reflect the problem of vertical vibration but it ignores the mutual coupling between the suspensions and the influence of the angular motion of the body in the pitch and roll directions on the comfort, and the control requirements for vehicle comfort cannot be fully described. The four-degrees-of-freedom model is often used to study the vertical jump of the front and rear suspensions and the body’s pitching motion. The seven-degrees-of-freedom model can fully reflect the vertical jump, pitch, and roll changes. Therefore, this paper selects the seven-degrees-of-freedom vehicle model as the research object, as shown in Figure 2.

The meanings of the symbols in Figure 2 are as follows: $m_{s}$ represents the sprung mass of the suspension, $z_{s}$ represents the vertical displacement at the body centroid, φ represents the vehicle roll angle, $I_{\varphi }$ represents the moment of inertia for mass roll angle on the spring of suspension, θ represents the vehicle pitch angle, $I_{\theta }$ represents the moment of inertia for mass pitch angle on the spring of suspension, $q_{1},q_{2},q_{3},q_{4}$ represent road excitation for wheels, $z_{u1},z_{u2},z_{u3},z_{u4}$ represent the vertical vibration displacement of the unsprung mass, $z_{s1},z_{s2},z_{s3},z_{s4}$ represent the vertical vibration displacement of the sprung mass, $u_{1},u_{2},u_{3},u_{4}$ represent the actuation force for the actuator, $c_{s1},c_{s2},c_{s3},c_{s4}$ represent the damping coefficient of the suspension damper, $k_{s1},k_{s2},k_{s3},k_{s4}$ represent the suspension spring stiffness, $k_{u1},k_{u2},k_{u3},k_{u4}$ represent the tire elasticity coefficient, $m_{u1},m_{u2},m_{u3},m_{u4}$ represent the unsprung mass, $L_{f}$ represents the distance from the mass center on the spring to the front axle, $L_{r}$ represents the distance from the mass center on the spring to the rear axle, $T_{f}$ represents the distance from the sprung mass center to the front wheel, $T_{r}$ represents the distance from the sprung mass center to the rear wheel. The seven degrees of freedom are $z_{s},\theta ,\varphi ,z_{u1},z_{u2},z_{u3},z_{u4}$.

When the pitch angle and roll angle are small, the dynamic differential equation of the seven degrees of freedom vehicle model is as follows:

Vertical displacements at the four endpoints of the body:

(1) $$\begin{array}{l} z_{s1}=z_{s}-L_{f}\theta -T_{f}\varphi \\ z_{s2}=z_{s}-L_{f}\theta +T_{f}\varphi \\ z_{s3}=z_{s}+L_{r}\theta -T_{r}\varphi \\ z_{s4}=z_{s}+L_{r}\theta +T_{r}\varphi \end{array}$$

Vertical motion at the center of body mass:

(2) $$m_{s}{\ddot{z}}_{s}=F_{s1}+F_{s2}+F_{s3}+F_{s4}+u_{1}+u_{2}+u_{3}+u_{4}$$

where $F_{si}$ is the resultant spring and damping force of the *i*-th suspension, $u_{i}$ is the actuation force of the *i*-th suspension, i=1,2,3,4.

Body pitching motion:

(3) $$I_{\theta }\ddot{\theta }=-L_{f}\sum_{i=1}^{2}F_{si}+L_{r}\sum_{i=3}^{4}F_{si}-L_{f}\sum_{i=1}^{2}u_{i}+L_{r}\sum_{i=3}^{4}u_{i}$$

Body roll motion:

(4) $$I_{\varphi }\ddot{\varphi }=(F_{s2}+u_{2}-F_{s1}-u_{1})T_{f}+(F_{s4}+u_{4}-F_{s3}-u_{3})T_{r}$$

Unsuspension mass vertical motion (four-wheel motion):

(5) $$\begin{array}{l} m_{u1}{\ddot{z}}_{u1}=k_{u1}(q_{1}-z_{u1})-u_{1}-F_{s1}\\ m_{u2}{\ddot{z}}_{u2}=k_{u2}(q_{2}-z_{u2})-u_{2}-F_{s2}\\ m_{u3}{\ddot{z}}_{u3}=k_{u3}(q_{3}-z_{u3})-u_{3}-F_{s3}\\ m_{u4}{\ddot{z}}_{u4}=k_{u4}(q_{4}-z_{u4})-u-4F_{s4} \end{array}$$

The resultant force of the spring and damper in the suspension:

(6) $$\begin{array}{l} F_{s1}=k_{s1}(z_{u1}-z_{s}+L_{f}\theta +T_{f}\varphi )+c_{s1}({\dot{z}}_{u1}-{\dot{z}}_{s}+L_{f}\dot{\theta }+T_{f}\dot{\varphi })\\ F_{s2}=k_{s2}(z_{u2}-z_{s}+L_{f}\theta -T_{f}\varphi )+c_{s2}({\dot{z}}_{u2}-{\dot{z}}_{s}+L_{f}\dot{\theta }-T_{f}\dot{\varphi })\\ F_{s3}=k_{s3}(z_{u3}-z_{s}-L_{r}\theta +T_{r}\varphi )+c_{s3}({\dot{z}}_{u3}-{\dot{z}}_{s}-L_{r}\dot{\theta }+T_{r}\dot{\varphi })\\ F_{s4}=k_{s4}(z_{u4}-z_{s}-L_{r}\theta -T_{r}\varphi )+c_{s4}({\dot{z}}_{u4}-{\dot{z}}_{s}-L_{r}\dot{\theta }-T_{r}\dot{\varphi }) \end{array}$$

Equation (2) is the acceleration term of the suspension sprung mass; Equation (3) is the pitch angular acceleration term of the suspension sprung mass; and Equation (4) is the roll angular acceleration term. They are all affected by the vibration displacement $z_{u1},z_{u2},z_{u3},z_{u4}$ of the wheel. Equation (6) is the dynamic equation of each wheel, and the vibration of each wheel is affected by the road surface excitation.

### 2.1. Dimension Reduction of System Model

Redefine the input variables for vertical motion (2), pitch motion (3), and roll motion (4) at the center of body mass, so that

(7) $$\begin{array}{l} u_{5}=\frac{1}{m_{s}}u_{1}+\frac{1}{m_{s}}u_{2}+\frac{1}{m_{s}}u_{3}+\frac{1}{m_{s}}u_{4}\\ u_{6}=-\frac{L_{f}}{I_{\theta }}u_{1}-\frac{L_{f}}{I_{\theta }}u_{2}+\frac{L_{r}}{I_{\theta }}u_{3}+\frac{L_{r}}{I_{\theta }}u_{4}\\ u_{7}=-\frac{T_{f}}{I_{\varphi }}u_{1}+\frac{T_{f}}{I_{\varphi }}u_{2}-\frac{T_{r}}{I_{\varphi }}u_{3}+\frac{T_{r}}{I_{\varphi }}u_{4} \end{array}$$

The 7-DOF vehicle model is organized as follows:

(8) $${\ddot{z}}_{u1}=a_{11}z_{u1}+a_{12}{\dot{z}}_{u1}-u_{1}/m_{u1}+h_{1}$$

(9) $${\ddot{z}}_{u2}=a_{21}z_{u2}+a_{22}{\dot{z}}_{u2}-u_{2}/m_{u2}+h_{2}$$

(10) $${\ddot{z}}_{u3}=a_{31}z_{u1}+a_{32}{\dot{z}}_{u1}-u_{3}/m_{u3}+h_{3}$$

(11) $${\ddot{z}}_{u4}=a_{41}z_{u1}+a_{42}{\dot{z}}_{u4}-u_{4}/m_{u4}+h_{4}$$

(12) $${\ddot{z}}_{s}=a_{51}z_{s}+a_{52}{\dot{z}}_{s}+u_{5}+h_{5}$$

(13) $$\ddot{\theta }=a_{61}\theta +a_{62}\dot{\theta }+u_{6}+h_{6}$$

(14) $$\ddot{\varphi }=a_{71}\varphi +a_{72}\dot{\varphi }+u_{7}+h_{7}$$

In the formula, $a_{11}=-\left(k_{u1}+k_{s1}\right)/m_{u1}$, $a_{12}=-c_{s1}/m_{u1}$, $a_{21}=-\left(k_{u2}+k_{s2}\right)/m_{u2}$, $a_{22}=-c_{s2}/m_{u2}$, $a_{31}=-\left(k_{u3}+k_{s3}\right)/m_{u3}$, $a_{32}=-c_{s3}/m_{u3}$, $a_{41}=-\left(k_{u4}+k_{s4}\right)/m_{u4}$, $a_{42}=-c_{s4}/m_{u4}$, $a_{51}=-\sum_{i=1}^{4}\frac{k_{si}}{m_{s}}$, $a_{52}=-\sum_{i=1}^{4}\frac{c_{si}}{m_{s}}$, $a_{61}=-\frac{L_{f}^{2}}{I_{\theta }}\sum_{i=1}^{2}k_{si}-\frac{L_{r}^{2}}{I_{\theta }}\sum_{i=3}^{4}k_{si}$, $a_{62}=-\frac{L_{f}^{2}}{I_{\theta }}\sum_{i=1}^{2}c_{si}-\frac{L_{r}^{2}}{I_{\theta }}\sum_{i=3}^{4}c_{si}$, $a_{71}=-\frac{T_{f}^{2}}{I_{\varphi }}\sum_{i=1}^{2}k_{si}-\frac{T_{r}^{2}}{I_{\varphi }}\sum_{i=3}^{4}k_{si}$, $a_{72}=-\frac{T_{f}^{2}}{I_{\varphi }}\sum_{i=1}^{2}c_{si}-\frac{T_{r}^{2}}{I_{\varphi }}\sum_{i=3}^{4}c_{si}$, $h_{1}=\frac{1}{m_{u1}}\left(k_{u1}q_{1}+k_{s1}z_{s}-k_{s1}L_{f}\theta -k_{s1}T_{f}\varphi +c_{s1}{\dot{z}}_{s}-c_{s1}L_{f}\dot{\theta }-c_{s1}T_{f}\dot{\varphi }\right)$, $h_{2}=\frac{1}{m_{u2}}\left(k_{u2}q_{2}+k_{s2}z_{s}-k_{s2}L_{f}\theta +k_{s2}T_{f}\varphi +c_{s2}{\dot{z}}_{s}-c_{s2}L_{f}\dot{\theta }+c_{s2}T_{f}\dot{\varphi }\right)$, $h_{3}=\frac{1}{m_{u3}}\left(k_{u3}q_{3}+k_{s3}z_{s}+k_{s3}L_{r}\theta -k_{s3}T_{r}\varphi +c_{s3}{\dot{z}}_{s}+c_{s3}L_{r}\dot{\theta }-c_{s3}T_{r}\dot{\varphi }\right)$, $h_{4}=\frac{1}{m_{u4}}\left(k_{u4}q_{4}+k_{s4}z_{s}+k_{s4}L_{r}\theta +k_{s4}T_{r}\varphi +c_{s4}{\dot{z}}_{s}+c_{s4}L_{r}\dot{\theta }+c_{s4}T_{r}\dot{\varphi }\right)$, $\begin{array}{l} h_{5}=\frac{1}{m_{s}}\sum_{i=1}^{4}\left(k_{si}z_{ui}+c_{si}{\dot{z}}_{ui}\right)+\frac{L_{f}}{m_{s}}\sum_{i=1}^{2}\left(k_{si}\theta +c_{si}\dot{\theta }\right)-\frac{L_{r}}{m_{s}}\sum_{i=3}^{4}\left(k_{si}\theta +c_{si}\dot{\theta }\right)\\ \;\;+\frac{T_{f}}{m_{s}}\sum_{i=1}^{2}{\left(-1\right)}^{i+1}\left(k_{si}\varphi +c_{si}\dot{\varphi }\right)+\frac{T_{r}}{m_{s}}\sum_{i=3}^{4}{\left(-1\right)}^{i+1}\left(k_{si}\varphi +c_{si}\dot{\varphi }\right) \end{array}$, $\begin{array}{l} h_{6}=\frac{L_{f}}{I_{\theta }}\sum_{i=1}^{2}\left[k_{si}\left(z_{s}-z_{ui}\right)+c_{si}\left({\dot{z}}_{s}-{\dot{z}}_{ui}\right)\right]+\frac{L_{r}}{I_{\theta }}\sum_{i=3}^{4}\left[k_{si}\left(z_{ui}-z_{s}\right)+c_{si}\left({\dot{z}}_{ui}-{\dot{z}}_{s}\right)\right]\\ \;+\frac{T_{f}L_{f}}{I_{\theta }}\sum_{i=1}^{2}{\left(-1\right)}^{i}\left(k_{si}\varphi +c_{si}\dot{\varphi }\right)+\frac{T_{r}L_{r}}{I_{\theta }}\sum_{i=3}^{4}{\left(-1\right)}^{i+1}\left(k_{si}\varphi +c_{si}\dot{\varphi }\right) \end{array}$, $\begin{array}{l} h_{7}=\frac{T_{f}}{I_{\varphi }}\sum_{i=1}^{2}{\left(-1\right)}^{i}\left[k_{si}\left(z_{ui}-z_{s}\right)+c_{si}\left({\dot{z}}_{ui}-{\dot{z}}_{s}\right)\right]+\frac{T_{r}}{I_{\varphi }}\sum_{i=3}^{4}{\left(-1\right)}^{i}\left[k_{si}\left(z_{ui}-z_{s}\right)+c_{si}\left({\dot{z}}_{ui}-{\dot{z}}_{s}\right)\right]\\ \;+\frac{L_{f}T_{f}}{I_{\varphi }}\sum_{i=1}^{2}{\left(-1\right)}^{i}\left(k_{si}\theta +c_{si}\dot{\theta }\right)+\frac{L_{r}T_{r}}{I_{\varphi }}\sum_{i=3}^{4}{\left(-1\right)}^{i+1}\left(k_{si}\theta +c_{si}\dot{\theta }\right) \end{array}$.

This paper selects the state vector of the system as $x_{1}={\left[\begin{array}{cc} z_{u1} & {\dot{z}}_{u1} \end{array}\right]}^{T}$, $x_{2}={\left[\begin{array}{cc} z_{u2} & {\dot{z}}_{u2} \end{array}\right]}^{T}$, $x_{3}={\left[\begin{array}{cc} z_{u3} & {\dot{z}}_{u3} \end{array}\right]}^{T}$, $x_{4}={\left[\begin{array}{cc} z_{u4} & {\dot{z}}_{u4} \end{array}\right]}^{T}$, $x_{5}={\left[\begin{array}{cc} z_{s} & {\dot{z}}_{s} \end{array}\right]}^{T}$, $x_{6}={\left[\begin{array}{cc} \theta & \dot{\theta } \end{array}\right]}^{T}$, $x_{7}={\left[\begin{array}{cc} \varphi & \dot{\varphi } \end{array}\right]}^{T}$, and system output as $y_{1}={\ddot{z}}_{u1}$, $y_{2}={\ddot{z}}_{u2}$, $y_{3}={\ddot{z}}_{u3}$, $y_{4}={\ddot{z}}_{u4}$, $y_{5}={\ddot{z}}_{s}$, $y_{6}=\ddot{\theta }$, $y_{7}=\ddot{\varphi }$. The control input is $u_{i}$. This paper mainly considers the control coupling and lists the remaining items as uncertain items $h_{i}$. The seven-DOF vehicle model (8)–(14) is abbreviated as follows:

(15) $${\dot{x}}_{i}=A_{i}x_{i}+B_{i}u_{i}+\omega_{i}h_{i}\;i=1,2,\cdots ,7$$

(16) $$y_{i}=C_{i}x_{i}+D_{i}u_{i}+\lambda_{i}h_{i}\;i=1,2,\cdots ,7$$

In the formula, $A_{i}=\left[0,1;a_{i1},a_{i2}\right]$, $C_{i}=\left[\begin{array}{cc} a_{i1} & a_{i2} \end{array}\right]$, $\lambda_{i}=1$, $B_{5}=B_{6}=B_{7}={\left[\begin{array}{cc} 0 & 1 \end{array}\right]}^{T}$, $\omega_{i}={\left[\begin{array}{cc} 0 & 1 \end{array}\right]}^{T}$, $D_{1}=-1/m_{u1}$, $D_{2}=-1/m_{u2}$, $D_{3}=-1/m_{u3}$, $D_{4}=-1/m_{u4}$, $D_{5}=D_{6}=D_{7}=1$, $B_{1}={\left[\begin{array}{cc} 0 & -1/m_{u1} \end{array}\right]}^{T}$, $B_{2}={\left[\begin{array}{cc} 0 & -1/m_{u2} \end{array}\right]}^{T}$, $B_{3}={\left[\begin{array}{cc} 0 & -1/m_{u3} \end{array}\right]}^{T}$, $B_{4}={\left[\begin{array}{cc} 0 & -1/m_{u4} \end{array}\right]}^{T}$.

It can be seen from Equations (15) and (16) that the 7-DOF vehicle model is decomposed into seven subsystems. According to the multi-agent theory, the seven subsystems can be regarded as seven agents, i=1,2,⋯,7. By designing the *i*-th agent control strategy, the system can follow the ideal value of its output.

### 2.2. System Control Model Based on Graph Theory

In multi-agent system graph theory, it is mainly composed of node sets and edge sets, represented by $G=\left(V,\kappa \right)$ [27]. Use $V=\left\{v_{1},v_{2},\cdots ,v_{n}\right\}$ to represent the node set, and define the node set as a finite non-empty set, where node set *V* contains *n* elements, and i∈{1,2,⋯,n} can be used to represent each node, representing *n* agents. Let $\kappa =\{\kappa_{1},\kappa_{2},\cdots \kappa_{m}\}$ denote the edge set. The edge $\varepsilon_{k}$ belonging to the edge set must have a corresponding node pair $\left(v_{i},v_{j}\right)$ in the node set *V*, where $v_{i}$ represents the start point and $v_{j}$ represents the end point. $A_{l}$ is the adjacency matrix, which represents the relationship between the subsystems in the system, element $a_{ij}$ is the relationship between the *i*-th agent and the *j*-th agent, the correlation is 1, and the non-correlation is 0, $D_{r}=diag(d_{1},d_{2},\cdots ,d_{n})$ is the In-degree matrix, among $d_{i}=\sum_{j=1,j\neq i}^{n}a_{ij}$, L is the Laplace matrix, $L=D_{r}-A_{l}$.

**Figure 3:**
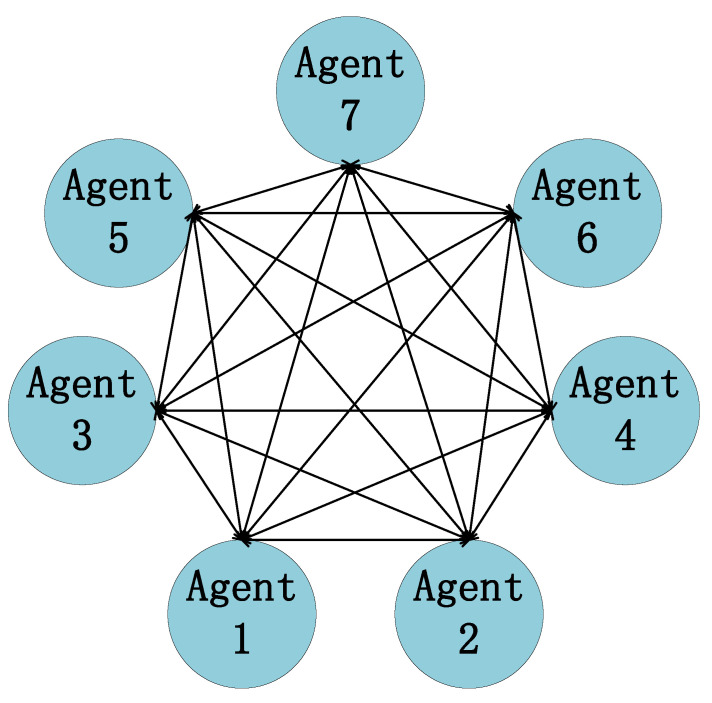
Multi-agent communication topology.

According to the communication topology and hardware connections of the seven subsystems (8)–(14), the topology of the active suspension control system based on graph theory is constructed as shown in Figure 3.

According to the basis of graph theory and matrix theory, the adjacency matrix of the seven agents $A_{l}$, In-degree matrix $D_{r}$, and Laplace matrix L is

$A_{l}=\left[\begin{array}{ccccccc} 0 & 1 & 1 & 1 & 1 & 1 & 1\\ 1 & 0 & 1 & 1 & 1 & 1 & 1\\ 1 & 1 & 0 & 1 & 1 & 1 & 1\\ 1 & 1 & 1 & 0 & 1 & 1 & 1\\ 1 & 1 & 1 & 1 & 0 & 1 & 1\\ 1 & 1 & 1 & 1 & 1 & 0 & 1\\ 1 & 1 & 1 & 1 & 1 & 1 & 0 \end{array}\right]$, $D_{r}=\left[\begin{array}{ccccccc} 6 & 0 & 0 & 0 & 0 & 0 & 0\\ 0 & 6 & 0 & 0 & 0 & 0 & 0\\ 0 & 0 & 6 & 0 & 0 & 0 & 0\\ 0 & 0 & 0 & 6 & 0 & 0 & 0\\ 0 & 0 & 0 & 0 & 6 & 0 & 0\\ 0 & 0 & 0 & 0 & 0 & 6 & 0\\ 0 & 0 & 0 & 0 & 0 & 0 & 6 \end{array}\right]$, $L=\left[\begin{array}{ccccccc} 6 & -1 & -1 & -1 & -1 & -1 & -1\\ -1 & 6 & -1 & -1 & -1 & -1 & -1\\ -1 & -1 & 6 & -1 & -1 & -1 & -1\\ -1 & -1 & -1 & 6 & -1 & -1 & -1\\ -1 & -1 & -1 & -1 & 6 & -1 & -1\\ -1 & -1 & -1 & -1 & -1 & 6 & -1\\ -1 & -1 & -1 & -1 & -1 & -1 & 6 \end{array}\right]$.

## 3. Distributed Model Predictive Control of Active Suspension for the i-th Agent

The goal of system control is to make the vertical displacement, pitch angle, and roll angle of the vehicle as small as possible, while at the same time reducing the acceleration of vibration in all directions. As a result, this paper presents a fast distributed model predictive control method for active suspension for engineering applications based on multi-agent theory and the concept of fast partial differential equation solving using neural networks.

### 3.1. The i-th Agent Prediction Model

The purpose of the control in this paper is to make the *i*-th agent output (16) quickly follow its ideal value under the condition of satisfying the state and control constraints.

The ideal values that define the output of the system are as follows:

(17) $$y_{i}^{*}=\rho_{i}x_{i}$$

In the formula, $\rho_{i}=\left[\begin{array}{cc} -k_{i1} & -k_{i2} \end{array}\right]$, i=1,2,⋯,7, according to the linear control theory, the necessary and sufficient condition for the stability of the second-order system is that each coefficient $k_{in}\left(i=1,2,\cdots ,7;n=1,2\right)$ of each system must be greater than zero. Selecting a large damping coefficient for the second-order system can significantly attenuate $z_{u1},z_{u2},z_{u3},z_{u4},z_{s},\theta ,\varphi$, but the selection of the coefficient must consider other indicators, such as the dynamic deflection of the suspension, the dynamic travel of the wheel, and so on. The simulation experiment [28] is used to determine $k_{i1}=0.25$, $k_{i2}=2$.

Let the output deviation $e_{yi}=y_{i}-y_{i}^{*}$, $\gamma_{i}=\rho_{i}-A_{i}$, according to Equations (16) and (17), we can obtain

(18) $$e_{yi}=\gamma_{i}x_{i}+D_{i}u_{i}+\lambda_{i}h_{i}$$

Definition P is the prediction time domain, m is the control time domain, and P≥m is defined. It is assumed that the control quantity outside the control time domain is unchanged, that is, $\Delta u_{i}\left(k+n\right)=0,n=m,m+1,\cdots ,P-1$. The indeterminate term does not change after time, which is $\Delta h_{i}\left(k+n\right)=0,n=1,2,\cdots ,P-1$.

Using the forward Euler method to discretize the state Equation (15) and output bias Equation (18) of the *i*-th agent, we can obtain

(19) $$x_{i}\left(k+1\right)=\left(I+TA_{i}\right)x_{i}\left(k\right)+TB_{i}u_{i}\left(k\right)+T\omega_{i}h_{i}\left(k\right)$$

(20) $$e_{yi}\left(k\right)=\gamma_{i}x_{i}\left(k\right)+D_{i}u_{i}\left(k\right)+\lambda_{i}h_{i}\left(k\right)$$

In the formula, *T* is the control period.

Write the discretized state Equation (19) and output deviation Equation (20) in the form of an incremental model:

(21) $$\Delta x_{i}(k+1)={\bar{A}}_{i}\Delta x_{i}(k)+{\bar{B}}_{i}\Delta u_{i}(k)+{\bar{\omega }}_{i}\Delta h_{i}(k)$$

(22) $$e_{yi}(k+1)=e_{yi}(k)+\gamma_{i}{\bar{A}}_{i}\Delta x_{i}(k)+\gamma_{i}{\bar{B}}_{i}\Delta u_{i}(k)+\gamma_{i}{\bar{\omega }}_{i}\Delta h_{i}(k)+D_{i}\Delta u_{i}(k+1)$$

In the formula, ${\bar{A}}_{i}=I+TA_{i}$, ${\bar{B}}_{i}=TB_{i}$, ${\bar{\omega }}_{i}=T\omega_{i}$, $\Delta x_{i}\left(k\right)=x_{i}\left(k\right)-x_{i}\left(k-1\right)$, $\Delta u_{i}(k)=u_{i}(k)-u_{i}(k-1)$, $\Delta h_{i}(k)=h_{i}(k)-h_{i}(k-1)$.

According to the incremental model of the system (21), we can obtain

(23) $$\begin{array}{l} \Delta x_{i}(k+2)={\bar{A}}_{i}\Delta x_{i}(k+1)+{\bar{B}}_{i}\Delta u_{i}(k+1)+{\bar{\omega }}_{i}\Delta h_{i}(k+1)\\ \;\;\;\;\;={\bar{A}}_{i}^{2}\Delta x_{i}(k)+{\bar{A}}_{i}{\bar{B}}_{i}\Delta u_{i}(k)+{\bar{A}}_{i}{\bar{\omega }}_{i}\Delta h_{i}(k)+{\bar{B}}_{i}\Delta u_{i}(k+1)\\ \Delta x_{i}(k+P)={\bar{A}}_{i}^{P}\Delta x_{i}(k)+{\bar{A}}_{i}^{P-1}{\bar{B}}_{i}\Delta u_{i}(k)+{\bar{A}}_{i}^{P-1}{\bar{\omega }}_{i}\Delta h_{i}(k)+{\bar{A}}_{i}^{P-2}{\bar{B}}_{i}\Delta u_{i}(k+1)\\ \;\;\;\;\;+\cdots +{\bar{A}}_{i}^{P-m}{\bar{B}}_{i}\Delta u_{i}(k+m-1) \end{array}$$

among them, i=1,2,⋯,7 is the state prediction at time k to time k+1.

Similarly, according to Formula (22), we can obtain

(24) $$\begin{array}{l} e_{yi}(k+2)=e_{yi}(k)+\left(\gamma_{i}{\bar{A}}_{i}^{2}+\gamma_{i}{\bar{A}}_{i}\right)\Delta x_{i}(k)+\left(\gamma_{i}{\bar{A}}_{i}{\bar{B}}_{i}+\gamma_{i}{\bar{B}}_{i}\right)\Delta u_{i}(k)\\ \;\;\;\;+\left(\gamma_{i}{\bar{A}}_{i}{\bar{\omega }}_{i}+\gamma_{i}{\bar{\omega }}_{i}\right)\Delta h_{i}(k)+\left(\gamma_{i}{\bar{B}}_{i}+D_{i}\right)\Delta u_{i}(k+1)+D_{i}\Delta u_{i}(k+2)\\ e_{yi}(k+P)=e_{yi}(k)+\sum_{n=1}^{P}\gamma_{i}{\bar{A}}_{i}^{n}\Delta x_{i}(k)+\sum_{n=1}^{P}\gamma_{i}{\bar{A}}_{i}^{n-1}{\bar{B}}_{i}\Delta u_{i}(k)+\sum_{n=1}^{P}\gamma_{i}{\bar{A}}_{i}^{n-1}{\bar{\omega }}_{i}\Delta h_{i}(k)\\ \;\;\;\;+\left(\sum_{n=1}^{P-1}\left(\gamma_{i}{\bar{A}}_{i}^{n-1}{\bar{B}}_{i}\right)+D_{i}\right)\Delta u_{i}(k+1)\\ \;\;\;\;+\cdots +\left(\sum_{n=1}^{P-m+1}\left(\gamma_{i}{\bar{A}}_{i}^{n-1}{\bar{B}}_{i}\right)+D_{i}\right)\Delta u_{i}(k+m-1) \end{array}$$

Define the P step prediction state vector, the output bias vector, and the m step input vector as follows:

(25) $$X_{P,i}\left(k\right)={\left[\begin{array}{cccc} \Delta x_{i}\left(k+1\right) & \Delta x_{i}\left(k+2\right) & \cdots & \Delta x_{i}\left(k+P\right) \end{array}\right]}^{T}$$

(26) $$E_{P,yi}(k)={\left[\begin{array}{cccc} e_{yi}\left(k+1\right) & e_{yi}\left(k+2\right) & \cdots & e_{yi}\left(k+P\right) \end{array}\right]}^{T}$$

(27) $$U_{i}\left(k\right)={\left[\begin{array}{cccc} \Delta u_{i}\left(k\right) & \Delta u_{i}\left(k+1\right) & \cdots & \Delta u_{i}\left(k+m-1\right) \end{array}\right]}^{T}$$

From Equations (23)–(27), the equations for predicting the next P steps of the system can be obtained:

(28) $$X_{P,i}\left(k\right)=\phi_{i}\Delta x_{i}\left(k\right)+\nu_{i}\Delta h_{i}\left(k\right)+\iota_{i}U_{i}\left(k\right)$$

(29) $$E_{P,yi}\left(k\right)=\xi_{i}e_{yi}\left(k\right)+\alpha_{i}\Delta x_{i}\left(k\right)+\varepsilon_{i}\Delta h_{i}\left(k\right)+\beta_{i}U_{i}\left(k\right)$$

In the formula, $\phi_{i}=\left[\begin{array}{c} {\bar{A}}_{i}\\ {\bar{A}}_{i}^{2}\\ \vdots \\ {\bar{A}}_{i}^{P} \end{array}\right]$, $\iota_{i}=\left[\begin{array}{ccccc} {\bar{B}}_{i} & 0 & 0 & \cdots & 0\\ {\bar{A}}_{i}{\bar{B}}_{i} & {\bar{B}}_{i} & 0 & \cdots & 0\\ \vdots & \vdots & \vdots & \ddots & \vdots \\ {\bar{A}}_{i}^{m-1}{\bar{B}}_{i} & {\bar{A}}_{i}^{m-2}{\bar{B}}_{i} & {\bar{A}}_{i}^{m-3}{\bar{B}}_{i} & \cdots & {\bar{B}}_{i}\\ \vdots & \vdots & \vdots & \ddots & \vdots \\ {\bar{A}}_{i}^{P-1}{\bar{B}}_{i} & {\bar{A}}_{i}^{P-2}{\bar{B}}_{i} & {\bar{A}}_{i}^{P-3}{\bar{B}}_{i} & \cdots & {\bar{A}}_{i}^{P-m}{\bar{B}}_{i} \end{array}\right]$, $\varepsilon_{i}=\left[\begin{array}{c} \gamma_{i}{\bar{\omega }}_{i}\\ \sum_{n=1}^{2}\gamma_{i}{\bar{A}}_{i}^{n-1}{\bar{\omega }}_{i}\\ \vdots \\ \sum_{n=1}^{P}\gamma_{i}{\bar{A}}_{i}^{n-1}{\bar{\omega }}_{i} \end{array}\right]$, $\alpha_{i}=\left[\begin{array}{c} \gamma_{i}{\bar{A}}_{i}\\ \sum_{n=1}^{2}\gamma_{i}{\bar{A}}_{i}^{n}\\ \vdots \\ \sum_{n=1}^{P}\gamma_{i}{\bar{A}}_{i}^{n} \end{array}\right]$, $\nu_{i}=\left[\begin{array}{c} {\bar{\omega }}_{i}\\ {\bar{A}}_{i}{\bar{\omega }}_{i}\\ \vdots \\ {\bar{A}}_{i}^{P-1}{\bar{\omega }}_{i} \end{array}\right]$, $\xi_{i}=\left[\begin{array}{c} I\\ I\\ \vdots \\ I \end{array}\right]$, $\beta_{i}=\left[\begin{array}{ccccc} \gamma_{i}{\bar{B}}_{i} & D_{i} & 0 & \cdots & 0\\ \sum_{n=1}^{2}\;\gamma_{i}{\bar{A}}_{i}^{n-1}{\bar{B}}_{i} & \gamma_{i}{\bar{B}}_{i} & D_{i} & \cdots & 0\\ \vdots & \vdots & \vdots & \ddots & \vdots \\ \sum_{n=1}^{m}\;\gamma_{i}{\bar{A}}_{i}^{n-1}{\bar{B}}_{i} & \sum_{n=1}^{m-1}\;\left(\gamma_{i}{\bar{A}}_{i}^{n-1}{\bar{B}}_{i}\right)+D_{i} & \cdots & \cdots & \gamma_{i}{\bar{B}}_{i}+D_{i}\\ \vdots & \vdots & \vdots & \ddots & \vdots \\ \sum_{n=1}^{P}\;\gamma_{i}{\bar{A}}_{i}^{n-1}{\bar{B}}_{i} & \sum_{n=1}^{P-1}\left(\gamma_{i}{\bar{A}}_{i}^{n-1}{\bar{B}}_{i}\right)+D_{i} & \cdots & \cdots & \sum_{n=1}^{P-m+1}\left(\gamma_{i}{\bar{A}}_{i}^{n-1}{\bar{B}}_{i}\right)+D_{i} \end{array}\right].$

### 3.2. Fast Rolling Optimization Based on RBF Neural Network

#### 3.2.1. Optimization Indicators

In order to improve the ride comfort and handling stability of the vehicle and reduce the loss of control energy, a multi-objective optimization function $J_{i}$ is defined.

Firstly, in order to improve the ride comfort and handling stability of the whole vehicle, the predicted output value of the system is made close to the ideal value under the constraints of the system state and control input.

According to the dimensionality reduction control model of active suspension based on graph theory, this paper considers the hardware connection and communication topology among seven agents, as well as the influence of other agents on their own agents, and the output following the deviation of the *i*-th agent is defined as $\psi_{i}(k)=\sum_{j=1,i\neq j}^{7}a_{ij}\left(E_{m,yi}\left(k\right)-E_{m,yj}\left(k\right)\right)$.

Define $J_{i1}$ as follows:

(30) $$J_{i1}(k)={\left\|\psi_{i}(k)\right\|}_{Q_{i}}^{2}=\psi_{i}^{T}(k)Q_{i}\psi_{i}(k)$$

In the formula, $E_{m,yi}\left(k\right)$ is the systematic deviation vector of the m step prediction, $a_{ij}$ is the element of the adjacency matrix $A_{l}$, $Q_{i}$ is the weight matrix, which represents the degree of tracking error suppression.

Secondly, in order to ensure the system stability of the proposed control method, a terminal error is introduced and $J_{i2}$ is defined as follows:

(31) $$J_{i2}\left(k\right)={\left\|e_{yi}\left(k+m\right)\right\|}_{F_{i}}^{2}$$

In the formula, $F_{i}$ is the weight matrix, which represents the degree of terminal error suppression.

Finally, in order to ensure that the control actions in the entire control process are within the allowable range to reduce energy loss, and considering the energy saving of the vehicle system, $J_{i3}$ is defined as follows:

(32) $$J_{i3}\left(k\right)={\left\|\Delta U_{i}\left(k+P\right)\right\|}_{R_{i}}^{2}$$

In the formula, $R_{i}$ is the weight matrix, which represents the inhibition degree of the control quantity.

Therefore, the optimization metric of the *i*-th agent is in the form

(33) $$\min J_{i}(k)=\min\left(J_{i1}(k)+J_{i2}(k)+J_{i3}(k)\right)$$

#### 3.2.2. Constraints

Firstly, to satisfy the dynamic constraints of the system

(34) $$\begin{array}{l} x_{i}(k+1)={\bar{A}}_{i}x_{i}(k)+{\bar{B}}_{i}u_{i}(k)+{\bar{\omega }}_{i}h_{i}(k)\\ e_{yi}\left(k\right)=\gamma_{i}x_{i}\left(k\right)+D_{i}u_{i}\left(k\right)+\lambda_{i}h_{i}\left(k\right) \end{array}$$

Secondly, the state constraints of the system need to be satisfied:

(35) $$x_{i,\min}\leq x_{i}\leq x_{i,\max}$$

Finally, it is necessary to ensure that the output of the seven agent controllers is within the allowable range, and the control constraints can be obtained according to Formula (7) as follows:

(36) $$\begin{array}{l} \;\;\;\;u_{i,\min}\leq u_{i}\leq u_{i,\max}\\ \;\;\;m_{s}u_{5}-u_{1}-u_{2}-u_{3}-u_{4}=0\\ I_{\theta }u_{6}+L_{f}u_{1}+L_{f}u_{2}-L_{r}u_{3}-L_{r}u_{4}=0\\ \;I_{\varphi }u_{7}+T_{f}u_{1}-T_{f}u_{2}+T_{r}u_{3}-T_{r}u_{4}=0 \end{array}$$

#### 3.2.3. Quadratic Programming Solution

According to the constraint Equations (34)–(36) and the performance index Equation (33), the optimization indicators in this paper are organized into the following standard quadratic programming problems:

(37) $$J_{QP,i}\left(k\right)=\Gamma_{i}+2M_{i}^{T}{\bar{Q}}_{i}\beta_{i}U_{i}+U_{i}^{T}\left(\beta_{i}^{T}{\bar{Q}}_{i}\beta_{i}+R_{i}\right)U_{i}-2\sum_{j=1;i\neq j}^{7}a_{ij}U_{j}^{T}\beta_{i}^{T}{\bar{Q}}_{i}\beta_{i}U_{i}$$

In the formula,


$${\bar{Q}}_{i}=diag\left(\begin{array}{cccc} Q & \cdots & Q & F \end{array}\right),$$

$$M_{i}\left(k\right)=\xi_{i}E_{yi}\left(k\right)+\alpha_{i}\Delta x_{i}\left(k\right)+\varepsilon_{i}\Delta h_{i}\left(k\right)-\sum_{j=1;i\neq j}^{7}a_{ij}\left(\xi_{j}E_{yj}\left(k\right)+\alpha_{j}\Delta x_{j}\left(k\right)+\varepsilon_{j}\Delta h_{j}\left(k\right)\right),$$

$$\Gamma_{i}\left(k\right)=M_{i}^{T}\left(k\right){\bar{Q}}_{i}M_{i}\left(k\right)-2M_{i}^{T}\left(k\right){\bar{Q}}_{i}\sum_{j=1;i\neq j}^{7}a_{ij}\beta_{i}U_{j}\left(k\right)+\sum_{j=1;i\neq j}^{7}a_{ij}U_{j}^{T}\left(k\right)\beta_{i}^{T}{\bar{Q}}_{i}\sum_{j=1;i\neq j}^{7}a_{ij}\beta_{i}U_{j}\left(k\right).$$

Since $M_{i}\left(k\right),\Gamma_{i}\left(k\right)$ in formula (37) has no relationship with $U_{i}\left(k\right)$, it does not affect the optimization of performance indicators and can be ignored.

In the process of a rolling optimization solution, the model prediction output in an analytical expression can be used with quadratic programming to solve the optimal control sequence:

(38) $$\frac{\partial J_{QP,i}}{\partial u_{i}}=2M_{i}^{T}{\bar{Q}}_{i}\beta_{i}+2\left(\beta_{i}^{T}{\bar{Q}}_{i}\beta_{i}+R_{i}\right)U_{i}-2\sum_{j=1;i\neq j}^{7}a_{ij}U_{j}\beta_{i}^{T}{\bar{Q}}_{i}\beta_{i}$$

(39) $$U_{i}^{*}={\left(\beta_{i}^{T}{\bar{Q}}_{i}\beta_{i}+R_{i}\right)}^{-1}\left(\sum_{j=1;i\neq j}^{7}a_{ij}U_{j}\beta_{i}^{T}-M_{i}^{T}\right){\bar{Q}}_{i}\beta_{i}$$

For the *i*-th agent, in the process of converting the standard quadratic programming problem, the control quantity $U_{j}$ of other *j*-th agents is replaced by the control input sequence $U_{j}(k|k-1)$ predicted at the previous moment.

So far, the parameterized MPC problem described in Equation (33) has been transformed into a standard quadratic programming problem, which can be directly solved by using the quadratic programming algorithm.

#### 3.2.4. Partial Differential Equation Solution Based on RBF Neural Network

The QP solution process involves the inverse operation of the solution matrix, which reduces the solution speed of MPC. It is difficult to realize engineering applications. The RBF neural network has a simple structure and strong nonlinear fitting ability. It has a global best approximation property. It can approximate any nonlinear function with arbitrary precision [29]. Therefore, to properly weigh the computational efficiency and dynamic performance index of the system, the rolling optimization of DMPC is optimized using the RBF neural network in this study.

In this paper, the RBF neural network is used to solve the partial differential equation shown in Equation (38). With $\eta_{i}={\left[\begin{array}{cccc} e_{yi}\left(k+1\right) & \cdots & e_{yi}\left(k+P\right) & \Delta x_{i}\left(k\right) \end{array}\right]}^{T}$ as the input of the network, the number of nodes from the input to the output of the network is m+1,l,m, respectively, and the output form of the system is

(40) $${\hat{U}}_{i}^{*}=\sigma \left(\eta_{n}\upsilon_{n1}+\mu_{n1}\right)\upsilon_{n2}+\mu_{n2}$$

For this neural network, the model parameters can be expressed as

(41) $$\left(\upsilon_{n}^{*},\mu_{n}^{*}\right)=\underset{\left(\upsilon_{n},\mu_{n}\right)}{\arg\min}J_{i}\left(\varsigma_{n}\right)$$

In the formula, $\varsigma_{n}$ represents the set of network parameters $\left\{\upsilon_{n},\mu_{n}\right\}$, and the optimization of parameters adopts stochastic gradient descent. The iterative formula is as follows:

(42) $$\varsigma_{n}^{\left(N+1\right)}=\varsigma_{n}^{\left(N\right)}-\tau \nabla_{\varsigma n}J_{i}\left(\varsigma_{n}^{\left(N\right)}\right)$$

In the formula, τ is the Nth iteration step size, and the gradient $\nabla_{\varsigma n}J$ of the loss function relative to the model parameters is usually calculated using backpropagation, which is a special case of the reverse mode automatic differentiation technique.

Using the data samples obtained in the model prediction as the input of the network, through the training of the neural network, the function that maps the input vector to the output vector can be found, and the solution of the optimal weight approximation equation can be found, so that ${\hat{U}}_{i}^{*}$ can be easily solved.

### 3.3. Feedback Mechanism

In the actual application process, the existence of external interference is inevitable, which will cause certain errors in the prediction model and result in a deviation of the predicted output value from the ideal value. Therefore, a feedback strategy will be added to the control system to correct the prediction. The combination of the model, rolling optimization, and feedback correction can make the prediction model closer to the actual situation and improve the anti-interference ability of the prediction model.

Select the first element $\Delta {\hat{u}}_{i}^{*}\left(k\right)$ in the predicted time domain control sequence ${\hat{U}}_{i}^{*}\left(k\right)={\left[\begin{array}{cccc} \Delta {\hat{u}}_{i}^{*}\left(k\right) & \Delta {\hat{u}}_{i}^{*}\left(k+1\right) & \cdots & \Delta {\hat{u}}_{i}^{*}\left(k+m-1\right) \end{array}\right]}^{T}$, let $u_{i}^{*}(k)=u_{i}^{*}(k-1)+\Delta {\hat{u}}_{i}^{*}(k)$. Apply $u_{i}^{*}\left(k\right)$ to the system as the input of the controller at the next moment, where i=1,2,⋯,7. Predict the output at the next moment according to the state quantity and perform error compensation through feedback correction, such as rolling optimization, to improve the control accuracy of the system.

## 4. Simulation Verification

**Figure 4:**
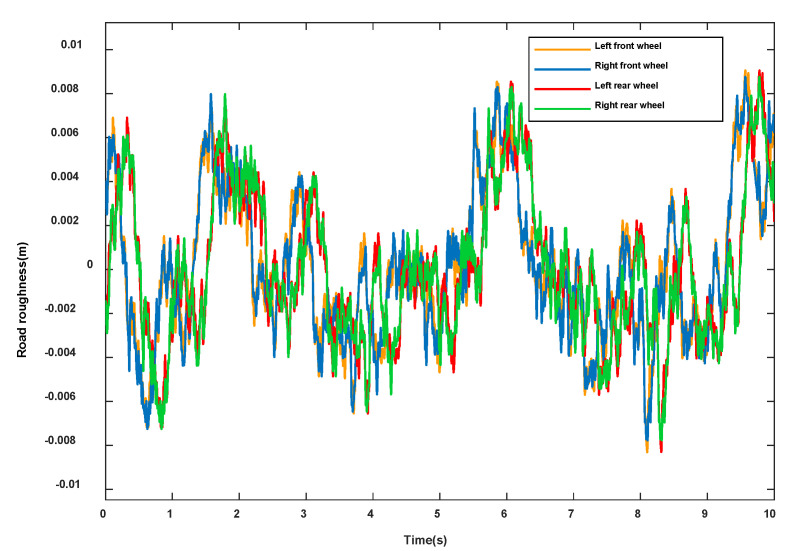
Pavement Incentives.

Establishing a road disturbance input model is the basis for studying vehicle dynamic response and its control [30]. In general, in order to ensure that the actual road surface is consistent with the obtained time domain road surface, $f_{0}=0.0628\mathrm{Hz}$.The four wheels are stimulated by the road surface, as shown in Figure 4. The excitation of the rear wheel and the front wheel of the car is time-delayed.

The vehicle 7-DOF model and the road excitation model were built in Matlab/Simulink, and the simulation was combined with Carsim. Under the B-level road excitation input, the simulation model runs at a constant speed of 72 km/h for 10 s. The parameters of the vehicle model selected in this paper are shown in Table 1.

**Figure 5:**
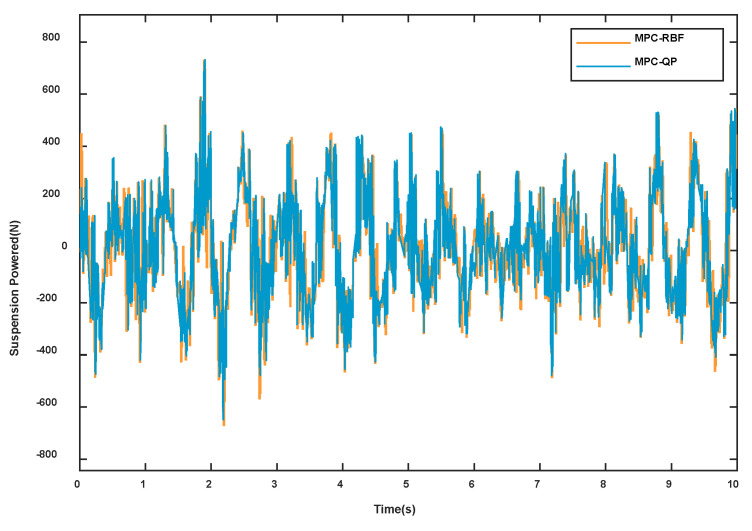
RBF neural network prediction result graph.

In order to verify the optimization effect and effectiveness of the RBF neural network modeling method, this paper uses nonlinear objects for simulation experiments and compares the accuracy of the RBF neural network combined with model predictive control and conventional model prediction (taking u1 as an example). The simulation results are shown in Figure 5.

**Figure 6:**
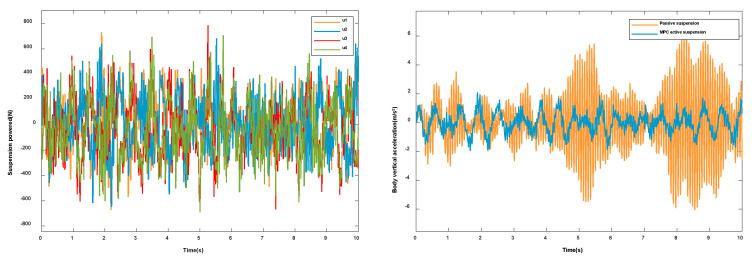

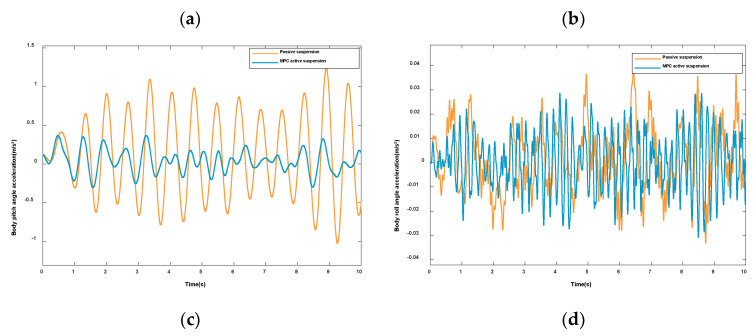
Vehicles under normal driving conditions: (**a**) Suspension actuation force. (**b**) Vertical acceleration. (**c**) Pitch angular acceleration. (**d**) Roll angular acceleration.

When the vehicle drives on the road at a constant speed, the body will shake to different degrees, which will affect the riding comfort and driving stability. According to the control algorithm proposed in this paper, the actuating forces acting on the four suspension agents are solved, as shown in Figure 6a. An uncontrolled suspension is introduced for comparison to reflect the improvement effect of the control strategy proposed in this paper on the ride comfort and handling stability of the vehicle. For ride comfort, the most intuitive evaluation index is to minimize the level of acceleration vibration felt by people. The simulation results are shown in Figure 6. When the four wheels are excited by the road surface, the vertical acceleration (Figure 6b), pitch angular acceleration (Figure 6c), and roll angular acceleration (Figure 6d) of the vehicle body are all greatly reduced.

**Figure 7:**
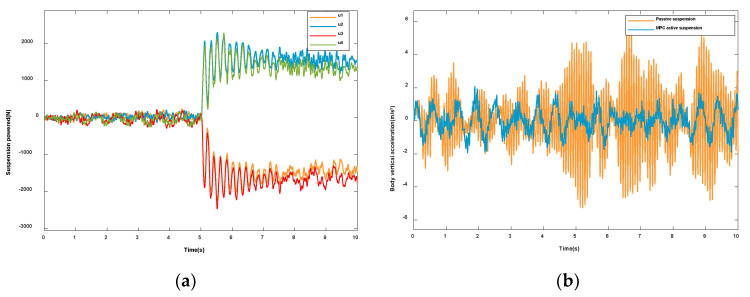

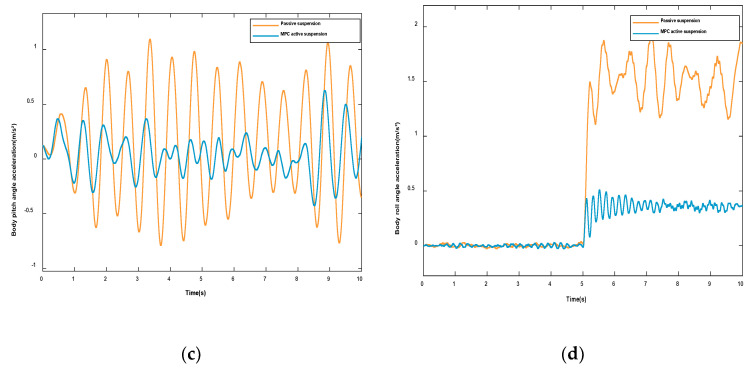
Vehicles under turning conditions: (**a**) Suspension actuation force. (**b**) Vertical acceleration. (**c**) Pitch angular acceleration. (**d**) Roll angular acceleration.

According to the simulation results in Figure 6, the vertical acceleration and pitch acceleration have been significantly improved. Since the roll effect is not obvious when the vehicle is driving at a constant speed on the B-level road, this paper chose to add steering at 5 s. According to the control algorithm proposed in this paper, the actuating forces acting on the four suspension agents are solved, as shown in Figure 7a. The simulation results under the steering condition are shown in Figure 7 Compared with the passive suspension, the control algorithm proposed in this paper is significantly lower in the vertical acceleration (Figure 7b), pitch angular acceleration (Figure 7c), and roll angular acceleration (Figure 7d). Therefore, it can be seen that, on the basis of reducing the vertical motion of the body, the algorithm also suppresses the pitching and rolling motions of the body and improves the riding comfort and driving stability of the vehicle.

**Figure 8:**
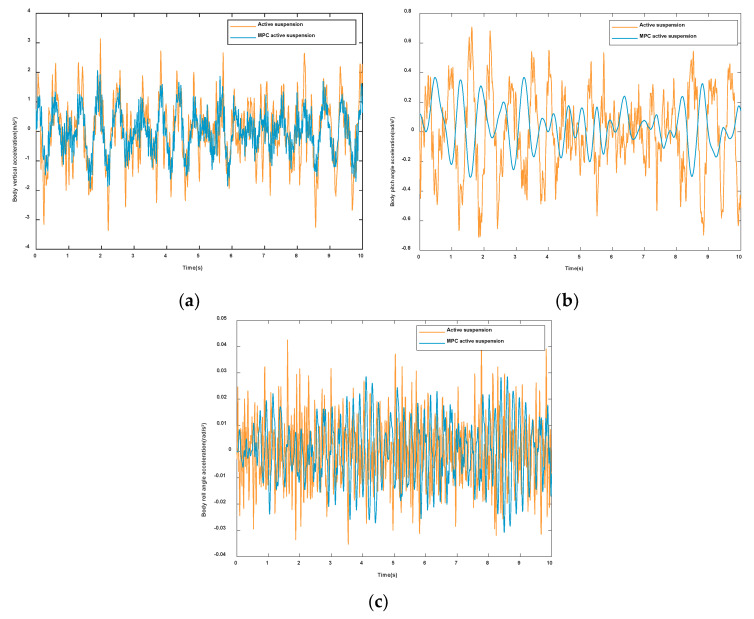
Comparing Results with Conventional Model Predictive Control: (**a**)Vertical acceleration. (**b**) Pitch angular acceleration. (**c**) Roll angular acceleration.

In this paper, the proposed control strategy is compared to the conventional model predictive control to verify its effectiveness in improving the ride comfort and handling stability of the vehicle. The simulation results are shown in Figure 8. It can be seen intuitively from the figure that the vertical acceleration (Figure 8a) and the pitch angular acceleration (Figure 8b) of the vehicle body are greatly reduced, and the roll angular acceleration (Figure 8c) has also been improved. It can be seen that the control strategy has achieved good effects on ride comfort and driving stability.

## 5. Conclusions

This paper establishes a seven-degrees-of-freedom vehicle model and uses the active suspension system as the research object. The performance index and computational effectiveness of the system are taken into consideration with the aim of reducing vertical acceleration, pitch angular acceleration, and roll angular acceleration. A fast-distributed-model-based predictive control strategy based on multi-agents is proposed, which comprehensively analyzes the suspension performance through multiple performance indicators. The proposed method is compared with passive suspension and conventional model prediction algorithms by using CarSim and Matlab/Simulink. The outcomes demonstrate that the control strategy suggested in this research has little impact on the scheme’s optimality. Additionally, the vertical acceleration, pitch angular acceleration, and roll angular acceleration of the vehicle body are significantly reduced, particularly in the steering condition, allowing for simultaneous consideration of the vehicle’s safety, comfort, and handling stability. The calculation results show that, compared with passive suspension, the vertical acceleration of the vehicle body, the pitch angle acceleration, and the roll angle acceleration of the proposed method are reduced by 47%, 54.2%, and 15.5%, respectively. Compared with conventional model prediction algorithms of active suspension, the vertical acceleration of the vehicle body, the pitch angle acceleration, and the roll angle acceleration of the proposed method are reduced by 32.6%, 33.7%, and 8.7%, respectively. This verifies the effectiveness of the control algorithm that was designed.

## Figures and Tables

**Table 1:** Parameters related to 7-DOF vehicles.

Parameter	Value	Unit
$m_{s}$	1370	kg
$m_{u}$	40	kg
$I_{\varphi }$	606	$\mathrm{kg}\cdot m^{2}$
$I_{\theta }$	4192	$\mathrm{kg}\cdot m^{2}$
$c_{s1,2}$	2228	N/(m/s)
$c_{s3,4}$	2210	N/(m/s)
$k_{s1,2}$	153	kN/m
$k_{s3,4}$	82	kN/m
$k_{u}$	230	kN/m
$L_{f}$	1.111	m
$L_{r}$	1.666	m
$T_{f}$	0.7525	m
$T_{r}$	0.7525	m

## Data Availability

Data are contained within the article.
